# Applying psychological theories to evidence-based clinical practice: identifying factors predictive of lumbar spine x-ray for low back pain in UK primary care practice

**DOI:** 10.1186/1748-5908-6-55

**Published:** 2011-05-28

**Authors:** Jeremy M Grimshaw, Martin P Eccles, Nick Steen, Marie Johnston, Nigel B Pitts, Liz Glidewell, Graeme Maclennan, Ruth Thomas, Debbie Bonetti, Anne Walker

**Affiliations:** 1 Clinical Epidemiology Programme, Ottawa Health Research Institute and Department of Medicine, University of Ottawa, 1053 Carling Avenue, Administration Building Room 2-017, Ottawa, K1Y 4E9, Canada; 2 Institute of Health and Society, Newcastle University, Baddiley-Clark Building, Richardson Road, Newcastle upon Tyne, NE2 4AX, UK; 3 College of Life Sciences and Medicine, University of Aberdeen, Health Sciences Building (2nd floor), Foresterhill, Aberdeen, AB25 2ZD, UK; 4 Dental Health Services & Research Unit, University of Dundee, MacKenzie Building, Kirsty Semple Way, Dundee, DD2 4BF, UK; 5 Leeds Institute of Health Sciences, University of Leeds, Charles Thackrah Building, 101 Clarendon Road, Leeds, LS2 9LJ, UK; 6 Health Services Research Unit, University of Aberdeen, Foresterhill, Aberdeen, AB25 2ZD, UK

## Abstract

**Background:**

Psychological models predict behaviour in a wide range of settings. The aim of this study was to explore the usefulness of a range of psychological models to predict the health professional behaviour 'referral for lumbar spine x-ray in patients presenting with low back pain' by UK primary care physicians.

**Methods:**

Psychological measures were collected by postal questionnaire survey from a random sample of primary care physicians in Scotland and north England. The outcome measures were clinical behaviour (referral rates for lumbar spine x-rays), behavioural simulation (lumbar spine x-ray referral decisions based upon scenarios), and behavioural intention (general intention to refer for lumbar spine x-rays in patients with low back pain). Explanatory variables were the constructs within the Theory of Planned Behaviour (TPB), Social Cognitive Theory (SCT), Common Sense Self-Regulation Model (CS-SRM), Operant Learning Theory (OLT), Implementation Intention (II), Weinstein's Stage Model termed the Precaution Adoption Process (PAP), and knowledge. For each of the outcome measures, a generalised linear model was used to examine the predictive value of each theory individually. Linear regression was used for the intention and simulation outcomes, and negative binomial regression was used for the behaviour outcome. Following this 'theory level' analysis, a 'cross-theoretical construct' analysis was conducted to investigate the combined predictive value of all individual constructs across theories.

**Results:**

Constructs from TPB, SCT, CS-SRM, and OLT predicted behaviour; however, the theoretical models did not fit the data well. When predicting behavioural simulation, the proportion of variance explained by individual theories was TPB 11.6%, SCT 12.1%, OLT 8.1%, and II 1.5% of the variance, and in the cross-theory analysis constructs from TPB, CS-SRM and II explained 16.5% of the variance in simulated behaviours. When predicting intention, the proportion of variance explained by individual theories was TPB 25.0%, SCT 21.5%, CS-SRM 11.3%, OLT 26.3%, PAP 2.6%, and knowledge 2.3%, and in the cross-theory analysis constructs from TPB, SCT, CS-SRM, and OLT explained 33.5% variance in intention. Together these results suggest that physicians' beliefs about consequences and beliefs about capabilities are likely determinants of lumbar spine x-ray referrals.

**Conclusions:**

The study provides evidence that taking a theory-based approach enables the creation of a replicable methodology for identifying factors that predict clinical behaviour. However, a number of conceptual and methodological challenges remain.

## Background

Healthcare systems and professionals fail to deliver the quality of care to which they aspire. Multiple studies internationally have observed evidence to practice gaps demonstrating that 30 to 40 percent of patients do not get treatments of proven effectiveness, and equally discouraging, up to 25 percent of patients receive unnecessary care that is potentially harmful [1-3]. Such evidence to practice gaps have significant adverse effects on the health and social welfare of citizens and economic productivity.

Lumbar spine imaging for low back pain in primary care settings is an example of an evidence to practice gap. Low back pain is an extremely common presentation in primary care. However, lumbar spine imaging in patients under 50 years is of limited diagnostic benefit within primary care settings [4]. Globally, clinical guidelines for the management of low back pain do not recommend routine imaging of patients with low back pain [4-8]. Furthermore, standard lumbar spine x-rays (the most common imaging modality used by UK primary care physicians) are associated with significant ionising radiation dosage. Despite this, lumbar spine x-rays are the fourth most common x-ray request from UK primary care physicians [9], with x-ray referrals continuing at the rate of 7 per 1000 patients per year [10]. We conducted a trial that found that for the majority of primary care physician requests, case note review could not identify appropriate indications for referral [10]. The trial also observed a reduction in lumbar spine x-rays of 20 percent without apparent adverse effects following the introduction of educational messages [10].

Recognition of evidence to practice gaps has led to increased interest in more active strategies to disseminate and implement evidence. Over the past two decades, a considerable body of implementation research has been developed [11]. This research demonstrates that dissemination and implementation interventions can be effective, but provides little information to guide the choice or optimise the components of such complex interventions in practice [12,13]. The effectiveness of interventions appears to vary across different clinical problems, contexts, and organizations. Our understanding of potential barriers and enablers to dissemination and implementation is limited and hindered by a lack of a 'basic science' relating to determinants of professional and organizational behaviour and potential targets for intervention [14]. The challenge for implementation researchers is to develop and evaluate a theoretical base to support the choice and development of interventions as well as the interpretation of implementation study results [15]. Despite recent increased interest in the potential value of behavioural theory to predict healthcare professional behaviour, relatively few studies have assessed this. A recent review by Godin *et al*. explored the use of social cognitive models to better understand determinants of health care professionals' intentions and behaviours [16]. They identified 72 studies that provided information on the determinants of intention, but only 16 prospective studies that provided information on the determinants of behaviour.

The current study, one part of the PRIME (PRocess modelling in ImpleMEntation research) study) [17], aimed to investigate the use of a number of psychological theories to explore factors associated with primary care physician lumbar spine x-ray referrals. Previous PRIME studies have used similar methods to explore factors associated with primary care physicians' use of antibiotics for sore throats and general dental practitioners' use of routine intra-oral x-rays and preventive fissure sealants [18-20]. Variables were drawn from the Theory of Planned Behaviour (TPB) [21], Social Cognitive Theory (SCT) [22], Operant Learning Theory (OLT) [23] (http://www.bfskinner.org/BFSkinner/Home.html, Implementation Intentions (II) [24], Common Sense Self-Regulation Model (CS-SRM) [25], and Weinstein's Stage Model termed the Precaution Adoption Process (PAP) [26,27]. These specific theories, which are described in detail elsewhere [28], were chosen because they predict behaviour but vary in their emphasis. Some focus on motivation, proposing that motivation determines behaviour, and therefore the best predictors of behaviour are factors that predict or determine motivation (*e.g.*, TPB). Some place more emphasis on factors that are necessary to predict behaviour in people who are already motivated to change (*e.g.*, II). Others propose that individuals are at different stages in the progress toward behaviour change, and that predictors of behaviour may be different for individuals at different stages (*e.g.*, PAP). The specific models used in this study were chosen for three additional reasons. First, they have been rigorously evaluated with patients or with healthy individuals. Second, they allow us to examine the influence on clinical behaviour of perceived external factors, such as patient preferences and organisational barriers and facilitators. Third, they all explain behaviour in terms of variables that are amenable to change.

The objective of this study was to identify those theories and the theoretical constructs that predicted clinical behaviour, behavioural simulation (as measured by the decisions made in response to five written clinical scenarios) and behavioural intention for lumbar spine x-ray referral.

## Methods

The methods of the study are described in detail elsewhere [17-20]. Briefly, this was a predictive study of the theory-based cognitions and clinical behaviours of primary care physicians; in this paper, we report data on primary care physicians' lumbar spine x-ray requests. Study participants were a random sample of primary care physicians selected from a list of all such physicians in selected regions of Scotland (Grampian, Tayside, Lothian) and north England (Durham, Newcastle and South Tees) by a statistician using a list of random sampling numbers. Data on theory-based cognitions (predictor measures) and two interim outcome measures (stated behavioural intention and behavioural simulation) were collected by postal questionnaire survey during the 12-month period to which the behavioural data related. Behavioural data were collected from routine data systems in the hospitals that primary care physicians reported as their referral centres for lumbar spine x-rays. Planned analyses explored the predictive value of theories and theory-based cognitions in explaining variance in the behavioural data.

### Predictor measures

Theoretically-derived measures were developed following standard operationalisation protocols wherever possible [21,29-33]. The cognition questions were developed from semi-structured interviews with 18 primary care physicians in Scotland and north England that lasted up to 60 minutes. The interviews use standard elicitation methods and covered physicians' views and experiences about managing patients with low back pain. Responses were used to create the questions measuring constructs. Five knowledge questions were developed by the study team based on issues for which there was good evidence. Table 1 provides a summary of the predictor measures used in this study (see also [28]); the instrument is available as Additional File 1. Unless otherwise stated, all questions were rated on a 7-point scale from 'strongly disagree' to 'strongly agree.' We aimed to include at least three questions per psychological construct.

**Table 1:** Summary of the explanatory measures

**Theory of Planned Behaviour (Ajzen, 1991)**	
*Constructs (number of questions)*	*Example Question(s)*
Behavioural intention (3)	I intend to refer patients with back pain for an X-ray as part of their management
Attitude: Direct (3); Indirect^a ^(8 behavioural beliefs (bb) multiplied by 8 outcome evaluations (oe). The score was the mean of the summed multiplicatives.)	Direct: In general, the possible harm to the patient of a lumbar spine X-ray is outweighed by its benefits; Indirect: In general, referring patients with back pain for an X-ray would reassure them (bb) x reassuring patients with back pain is (oe: un/important)
Subjective Norm: Indirect (4 normative beliefs (nb) multiplied by 4 motivation to comply (mtc) questions. The score was the mean of the summed multiplicatives).	I feel under pressure from the NHS not to refer patients for an X-ray (nb) x How motivated are you to do what the NHS thinks you should (mtc: very much/not at all)
Perceived Behavioural Control: Direct (4); Indirect/power (14)^c^	Direct: Whether I refer patients for a lumbar X-ray is entirely up to me. **Indirect**: Without an X-ray, how confident are you in your ability to treat patients with back pain who expect me to refer them for an X-ray
**Social Cognitive Theory (Bandura,1998)**	
Risk Perception (3)	It is highly likely that patients with back pain will be worse off if I do not refer them for an X-ray.
Outcome Expectancies Self (2x2), Behaviour (8x8). The score was the mean of the summed multiplicatives.	Self: If I refer a patient with back pain for an X-ray, then I will think of myself as a competent GP x Thinking of myself as a competent GP is (Un/Important) *Behaviour*: See Attitude (Theory of Planned Behaviour)
Self Efficacy: General: Generalized Self-Efficacy Scale (Schwarzer, 1992) (10: 4 point scale, not at all true/exactly true); Specific (7)	*General*: I can always manage to solve difficult problems if I try hard enough *Specific*: How confident are you in your ability to treat back problems without using an X-ray report
**Implementation Intention (Gollwitzer, 1993)**	
Action planning (3)	Currently, my standard method of managing patients with back pain does not include referring them for an X-ray
**Operant Learning Theory (Skinner, Blackman, 1974)**	
Anticipated consequences (3)	If I start routinely referring patients with back pain then, on balance, my life as a GP will be easier in the long run
Evidence of habit (2)	When I see a patient with back pain, I automatically consider referring them for an X-ray
Experienced (rewarding and punishing) consequences (4: more likely to refer (score = 1); less likely (score=-1); unchanged/not sure/never occurred (score = 0)). Scores were summed.	Think about the last time you referred a patient for a lumbar spine X-ray and felt pleased that you had done so. Do you think the result of this episode has made you: Think about the last time you decided not to refer a patient for a lumbar spine X-ray and felt sorry that you had not done so. Do you think the result of this episode has made you:
**Common Sense Self-regulation Model**^**d **^**(Leventhal et al., 1984)**	
Perceived identity (3)	Back pain as seen in general practice is generally of an intense nature
Perceived cause (8)	Back pain is caused by stress or worry
Perceived controllability (7)	What the patient does can determine whether back pain gets better or worse, What I do can determine whether the patient's back pain gets better or worse
Perceived duration (5)	Back pain as seen in general practice is very unpredictable
Perceived consequences (3)	Back pain does not have much effect on a patient's life
Coherence (2)	I have a clear picture or understanding of back pain
Emotional response (4)	Seeing patients with back pain does not worry me
**Precaution Adoption Process (Stage model)(Weinstein, 1988; Weinstein, Rothman & Sutton, 1998)**	
Current stage of change. A single statement is ticked to indicate the behavioural stage	Unmotivated (3): I have not yet thought about changing the number of lumbar X-rays I currently request. It has been a while since I have thought about changing the number of lumbar X-rays I request. Motivated (2): I have thought about it and decided that I will not change the number of lumbar X-rays I request. I have decided that I will request more lumbar X-rays. I have decided that I will request less lumbar X-rays. Action (1): I have already done something about increasing the number of lumbar X-rays I request I have already done something about decreasing the number of lumbar X-rays I request
**Other Measures**	
Knowledge (5) (True/False/Not Sure)	The presence of spondolytic changes on a lumbar spine X-ray correlates well with back pain
Demographic	Post code, gender, time qualified, number of other doctors in practice, trainer status, hours per week, list size

^a ^All indirect measures consist of specific belief questions identified in the preliminary study as salient to the management of low back pain.

^b ^These individuals and groups were identified in the preliminary study as influential in the management of low back pain

^c ^An indirect measure of perceived behavioural control usually would be the sum of a set of multiplicatives (control beliefs x power of each belief to inhibit/enhance behaviour). However, the preliminary study demonstrated that it proved problematic to ask clinicians meaningful questions which used the word 'control' as clinicians tended to describe themselves as having complete control over the final decision to perform the behaviour. Support for measuring perceived behavioural control using only questions as to the ease or difficulty of performing the outcome behaviour was derived from a metanalysis which suggested that perceived ease/difficulty questions were sensitive predictors of behavioural intention and behaviour (Trafimow et al., 2002).

^d ^Illness representation measures were derived from the Revised Illness Perception Questionnaire (Moss-Morris, R., Weinman, J., Petrie, K. J., Horne, R., Cameron, L.D., & Buick, D. 2002)

### Outcome measures

#### Behaviour

The number of lumbar spine x-ray imaging requests made by each primary care physician over 12 months were obtained from the hospitals that the responding primary care physicians identified as their radiology referral centres. At the time of the study, primary care physicians in the United Kingdom did not have open access to other modalities of lumbar imaging (CT and MRI scans). We standardised our behaviour by the number of patients registered with the primary care doctor to reflect differences in workloads of the participating primary care doctors.

#### Behavioural simulation

Our measure used vignettes to simulate clinical decision-making in specific situations; such measures have been shown to be predictive of behaviour, though less so than general measures of intention [34]. Key elements which may influence primary care physicians' decisions to refer for a lumbar spine x-ray on patients with low back pain were identified from the literature, opinion of the clinical members of the research team, and the interviews with primary care physicians. From this, five clinical scenarios were constructed describing patients presenting in primary care with low back pain. Respondents were asked to decide whether or not they would request a lumbar spine x-ray for each scenario, and decisions to request an x-ray were summed to create a total score out of a possible maximum of five.

#### Behavioural intention

Three questions assessed primary care physicians' intention to refer patients presenting with low back pain for lumbar spine x-ray:

'When a patient presents with back pain, I have in mind to refer them for X-ray, I intend to refer patients with back pain for an X-ray as part of their management, I aim to refer patients with back pain for an X-ray as part of patient management (rated on a 7-point scale from 'Strongly Disagree' to 'Strongly Agree').'

Responses were summed (range 3 to 21) and scaled so that a low score equated with a low intention to refer for lumbar spine x-ray.

### Procedure

Participants were mailed an invitation pack (letter of invitation, questionnaire consisting of psychological and demographic measures, a form requesting consent to allow the research team to access the respondent's referral data, a study newsletter, and a reply paid envelope) by research staff. Initially, 700 primary care physicians were surveyed between July and mid-August 2003. Due to a low initial response rate, a further sample of 400 primary care physicians were surveyed between October and December 2003. Two postal reminders were sent to non-responders at two and four weeks. Behavioural data were collected over a one-year period, from approximately six months before to six months after the assessment of cognitions.

### Sample size and statistical analysis

The target sample size of 200 was based on a recommendation by Green [35] to have a minimum of 162 subjects when undertaking multiple regression analysis with 14 predictor variables.

The internal consistency of the measures was tested using Cronbach's alpha. If this was less than 0.6, then questionnaire items were removed from each measure to achieve the highest Cronbach's alpha possible. For constructs with only two questions, a correlation coefficient of 0.25 was used as a cut off.

For each of the three outcome variables, we examined the relationship between predictor and outcome variables within the structure of each of the theories individually. Spearman's correlation (for behaviour outcome) and Pearson Correlation Coefficients (for behavioural simulation and intention outcomes) between the individual constructs and the outcome measures were calculated. Given the distribution of the behavioural data, we used negative binomial regression (NBR) to model the predictive ability of individual theoretical constructs and complete theories. NBR is used to model count exhibiting over dispersion, as in the case of the behaviour outcome data in this study. We reported incidence rate ratios (IRR) from the NRB models. IRRs estimate the change in the rate of the dependent variable associated with changes in the independent variables. NBR does not generate a direct equivalent of an R^2 ^statistic to estimate the proportion of variance in the dependent variable explained by models. However, it is possible to compute a number of different R^2 ^statistics to explore the goodness of fit of the model [36]. The pseudo-R^2 ^we chose to use was McFaddens' adjusted R^2 ^because it penalizes models in the spirit of adjusted R^2 ^in linear regression for adding more variables to a model (see Additional File 2 for further discussion). Linear regression was used for intention and behavioural simulation. For the five 'perceived cause of illness' questions in the CS-SRM, responses were dichotomized into scores of five to seven (indicating agreement that the cause in question was responsible for low back pain) versus anything else (indicating disagreement). These dichotomous variables were then entered as independent variables into the regression models. The relationship between II and intention was not explored as it is a post-intentional theory. For the analysis of the PAP, respondents were dichotomized into two groups (decided to reduce or have already reduced x-rays versus other responses) and the relationship between predictive and outcome variables were examined using regression models. Finally, for predictors p < 0.25 irrespective of whether or not they came from the same theory, we conducted a cross-theoretical construct analyses that examined the relationship between predictive and outcome variables.

### Ethics approval

The study was approved by the UK South East Multi-Centre Research Ethics Committee (MREC/03/01/03).

## Results

**Figure 1:**
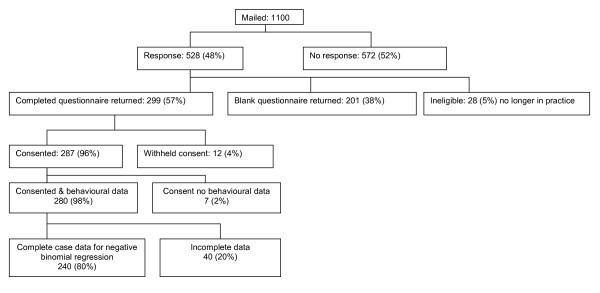
**Response rates**.

Of the 1,100 primary care physicians approached, 299 (27%) agreed to participate. Most respondents provided usable data on intention (296) and behavioural simulation (297), and we were able to obtain imaging request data from 287 (Figure 1). Numbers included in analyses vary between the outcome measures because complete case analysis was used. For the negative binomial regression analyses, we had complete data from 240 respondents.

Fifty eight percent of the respondents were male. Respondents had been qualified for a mean (SD) of 21 (8) years. They had a median inter-quartile range (IQR) list size of 1,450 registered patients, a median IQR of 4.8 (3.6 to 6.8) partners, and worked a median IQR of 8 (6 to 9) half day sessions a week; 45 (15%) were trainers. Descriptive statistics for the independent variables are provided in Table 2.

**Table 2:** Descriptive statistics

Theory	Predictive Constructs	N	Alpha	Mean	(SD)	Respondents agreeing with item (%)
Theory of	Attitude direct	2	0.25	4.6	(1.2)	
Planned	Attitude indirect	4	0.75	18.6	(6.9)	
Behaviour	Subjective Norm	4	0.68	15.0	(4.8)	
	
	Intention	3	0.69	2.1	(1.0)	
	PBC direct	4	0.63	4.5	(1.1)	
	PBC power	14	0.91	3.1	(1.0)	

Social Cognitive Theory	Risk perception	2	0.46	2.2	(1.0)	
	Outcome expectancies	6	0.76	13.9	(8.3)	
	Self efficacy	14	0.93	3.2	(0.8)	
	Generalised self efficacy	10	0.87	2.8	(0.4)	

Implementation Intention	Action Planning	-	-	2.4	(1.6)	

Operant Learning Theory	Anticipated consequences	2	0.46	2.2	(1.0)	
	Evidence of habitual behaviour	2	0.60	3.3	(1.7)	

Common Sense	Identity of condition	3	0.49	4.2	(0.8)	
Self-regulation	Timeline acute	2	0.19	3.4	(0.8)	
Model	Timeline cyclical	3	0.54	4.4	(0.9)	
	Control - by treatment	3	0.66	5.6	(0.8)	
	Control - by patient	2	0.85	5.7	(1.0)	
	Control - by doctor	2	0.36	5.3	(0.9)	
	Cause - stress	1				126 (42)
	Cause - family problems	1				117 (39)
	Cause - poor prior medical care	1				66 (22)
	Cause - patient's own behaviour	1				225 (85)
	Cause - ageing	1				217 (73)
	Cause - bad luck	1				140 (47)
	Cause - overwork	1				148 (49)
	Consequence	2	0.21	4.8	(0.8)	
	Emotional Response	4	0.69	5.1	(1.0)	
	Coherence	2	0.74	2.7	(1.0)	

Precaution Adoption Process						157 (53)†

Other	Knowledge	5	0.21	3.1	(1.0)	

*p≤0.05; ** p≤0.01; ***p≤0.001.

Alpha = Cronbach's

^†^Number of respondents who replied 'I have decided that I will request less lumbar X-rays' or 'I have already done something about decreasing the number of lumbar X-rays I request.'

### Relationship between the three outcome measures

The three outcome measures were significantly (though weakly) correlated with each other: for behaviour and behavioural simulation, the Spearman's rho statistic was 0.169 (p = 0.004); similarly for behaviour and behavioural intention it was 0.165 (p = 0.005); and for behavioural simulation and behavioural intention the Pearson's r was 0.313 (p < 0.001).

### Predicting behaviour

The mean number of lumbar spine x-rays was 5.0 per 1,000 patients registered per year. The results of analyses are shown in Table 3. Individual construct analyses suggested that constructs from TPB (attitudes, intention, and perceived behavioural control), SCT (risk perception, self efficacy), OLT (anticipated consequences) and CS-SRM (cause - aging) significantly predicted the lumbar spine referrals. To aid interpretation of the results, we provide the following example; intention had a mean score of 2.1 (SD 1.0), the IRR was 1.29 -- this suggests that for every point increase in intention (equivalent in this example to one SD), lumbar spine referrals would increase by 29.0%. Theory-level analyses (Table 3) suggested that TPB (perceived behavioural control), SCT (risk perception), OLT (anticipated consequences), CS-SRM (control - by patient, cause - poor prior medical care, cause - patients' own behaviours, cause - aging) predicted behaviour. II, PAP, and knowledge did not predict behaviour. However, the goodness to fit measures suggested that the theoretical models did not predict behaviour data in this dataset (McFadden's pseudo R^2 ^range from 0 to 0.004, see also Additional file 2 for addition goodness to fit measures). In the cross-theoretical construct analysis, constructs from TPB (attitudes) and CS-SRM (coherence, cause - poor prior medical care, control - by patient) were retained in the regression model; again the goodness of fit models performed poorly (Table 4).

**Table 3:** Predicting behaviour by psychological theory: negative binomial regression analyses

Theory	Predictive Constructs	IRR Individual and p-value		IRR model	
Theory of Planned	Intention	1.285	0.008	1.097	
Behaviour	PBC direct	1.023	0.823	1.175	
	PBC power	1.427	< 0.001	1.444**	R^2 ^= 0.004

Social Cognitive Theory	Risk perception	1.444	< 0.001	1.392**	
	Outcome expectancies	1.019	0.080	1.001	
	Self efficacy	1.363	0.019	1.110	
	Generalised self efficacy	0.855	0.564	0.823	R^2 ^= 0.002

Implementation Intention		1. 111	0.138	1.111	R^2 ^= 0.000

Operant Learning Theory	Anticipated consequences	1.449	< 0.001	1.413**	
	Evidence of habitual behaviour	1.089	0.179	1.017	R^2 ^= 0.004

Common Sense	Identity of condition	0.864	0.278	0.867	
Self-regulation	Timeline acute	1.08	0.957	1.026	
Model	Timeline cyclical	1.187	0.196	1.273	
	Control - by treatment	1.105	0.970	1.170	
	Control - by patient	0.869	0.142	0.725*	
	Control - by doctor	0.936	0.524	1.064	
	Cause - stress	1.191	0.370	0.519	
	Cause - family problems	1.345	0.130	2.526	
	Cause - poor prior medical care	1.403	0.134	1.70*	
	Cause - patient's own behaviour	0.897	0.581	0.592*	
	Cause - ageing	1.609	0.028	1.671*	
	Cause - bad luck	0.712	0.080	0.759	
	Cause - overwork	0.878	0.502	0.969	
	Consequence	1.006	0.902	1.060	
	Emotional Response	0.962	0.699	1.005	
	Coherence	1.231	0.046	1.171	R^2 ^= 0.000

Precaution Adoption Process		0.871	0.599	0.871	R^2 ^= 0.000

Knowledge		0.859	0.104	0.859	R^2 ^= 0.000

*p ≤ 0.05; ** p ≤ 0.01; ***p ≤ 0.001.

Alpha = Cronbach's; IRR Individual = incidence rate ratio from a regression model with the single construct independent variable IRR Model = incidence rate ratio from the theoretical model with all constructs included as independent variables. R^2 ^is MacFadden's adjusted R^2^.

**Table 4:** Results of the stepwise regression cross-theoretical construct analyses

Predictive Constructs	Entered				
**Outcome: Ordering lumbar spine x-rays**		**IRR**	**Adj. R**^**2**^		

**TPB**: Attitude Indirect and Direct; PBC Power; Intention **SCT**: Risk Perception; Self Efficacy **Operant learning theory**: anticipated consequences; Evidence of habitual behaviour **Implementation Intention** **CS-SRM **Timeline cyclical; Control - by patient; Cause - family problems, poor prior medical care, ageing, bad luck; Coherence **Knowledge**	Coherence	1.122*			
	
	Control - by patient	0.897*			
	
	Attitude Direct	1.017***			
	
	Cause - poor prior medical care	1.848**	0.015†		
	
					

**Outcome: Behavioural Simulation**		**Beta**	**Adj. R**^**2**^	**df**	**F**

**TPB**: Attitude Indirect and Direct; PBC Power and PBC Power direct; Intention **SCT**: Risk Perception; Outcome expectancy Self Efficacy **Operant learning theory**: Anticipated Consequences; Evidence of Habitual Behaviour **Implementation Intention** **CS-SRM**: Control - by treatment, patient, doctor; Cause - ageing; Coherence; Emotional Response **Precaution Adoption Process**	Action Planning	0.272***			
	
	PBC Power	0.252***			
	
	Cause - ageing	0.126*	0.165	3, 277	19.4***
	
					

**Outcome: Behavioural Intention**		**Beta**	**Adj. R**^**2**^	**df**	**F**

**TPB**: Attitude Indirect and Direct; Subjective Norm; PBC Power and PBC Power direct **SCT**: Risk Perception; Outcome expectancy Self Efficacy **Operant learning theory**: anticipated consequences; Evidence of Habitual Behaviour **CS-SRM**: Control - by treatment, patient and doctor; Cause- stress; Coherence; Emotional Response **Precaution Adoption Process** **Knowledge**	PBC Power	0.273***			
	
	Evidence of Habitual Behaviour	0.286***			
	
	Outcome expectancy	0.169**			
	
	Control - by treatment	-0.115*	0.335	4, 275	36.1***

*p ≤ 0.05; ** p ≤ 0.01; ***p ≤ 0.001.

PBC = perceived behavioural control; TPB = Theory of Planned Behaviour; SCT = Social Cognitive Theory; CS-SRM = Common Sense Self-Regulation Model;

† McFadden's pseudo R^2^

### Predicting behavioural simulation

In response to the five clinical scenarios, the respondents indicated that they would refer for lumbar spine x-ray in a mean (SD) of 1.5 (1.2) cases. The median number of referrals was 1 with a range of 0 to 3. From Table 5, the individual constructs that predicted behavioural simulation (*i.e.*, what primary care physicians said they would do in response to the specific clinical scenarios) were: TPB (attitudes, social norms, perceived behavioural control, and intention), SCT (risk perception, outcome expectancies, and self efficacy); II; OLT (anticipated consequences, evidence of habitual behaviour); CS-SRM (control - by treatment, control - by patient, control - by doctor, cause - ageing, emotional response treatment). Neither knowledge nor PAP predicted behavioural simulation.

**Table 5:** Predicting behavioural simulation and intention by psychological theory: correlation and multiple regression analyses

		Behavioural simulation					Behavioural intention				
**Theory**	**Predictive Constructs**	**r**	**Beta**	**R2(adj)**	**df**	**F**	**r**	**Beta**	**R2(adj)**	**df**	**F**

*Theory of Planned*	Intention	0.313***	0.182**								
*Behaviour*	PBC direct	-0.143*	0.018								
	PBC power	0.315***	0.236**	.116	3, 282	13.4***					
	
	Attitude direct						-0.180**	-0.088			
	Attitude indirect						0.361***	0.013			
	Subjective Norm						0.149**	-0.003			
	PBC direct						-0.320***	-0.068			
	PBC power						0.487***	0.090***	.250	5, 282	20.1***

*Social Cognitive*	Risk perception	0.286***	0.204**				0.392***	0.226***			
*Theory*	Outcome expectancies	0.139*	-0.023				0.350***	0.210**			
	Self efficacy	0.301***	0.245***				0.336***	0.197**			
	Generalised self efficacy	-0.036	-0.001	.121	4, 272	10.5***	-0.035	0.022	.215	4, 271	19.8***

*Implementation intention*		.135*	.135*	.015	1, 275	5.1*					

*Operant Learning Theory*	Anticipated consequences	0.286***	0.253***				0.392***	0.238***			
	Evidence of habitual behaviour	0.184**	0.080	.081	2, 287	13.7***	0.470***	0.371***	.263	2, 286	52.3***

*Common sense*	Identity of condition	-0.043	-0.029				0.043	0.081			
*Self regulation model*	Timeline acute	0.079	-0.029				0.097	0.000			
	Timeline cyclical	0.010	0.006				-0.020	-0.050			
	Control - by treatment	-0.187*	-0.115				-0.217**	-0.160**			
	Control - by patient	-0.121*	-0.004				-0.282**	-0.089			
	Control - by doctor	-0.140*	-0.024				-0.315**	-0.107			
	Cause - stress	-0.104	-0.051				-0.119*	-0.190			
	Cause - family problems	-0.096	-0.097				-0.080	0.084			
	Cause - poor prior medical care	0.039	0.100				-0.033	0.011			
	Cause - patient's own behaviour	0.040	0.074				-0.048	0.017			
	Cause - ageing	0.145***	0.145*				0.073	0.062			
	Cause - bad luck	0.053	0.071				-0.010	-0.044			
	Cause - overwork	-0.032	-0.080				0.046	0.052			
	Consequence	-0.080	-0.063				-0.061	-0.015			
	Emotional Response	-0.184***	-0.117				0.187**	-0.001			
	Coherence	0.089	-0.060	.036	16,268	1.7	-0.249**	-0.142**	.113	16,265	3.2***

*Precaution Adoption Process*		-0.09	-0.09	.005	1, 296	2.5	-0.17**	-0.17**	0.026	1, 294	8.3**

*Knowledge*		-.091	-.091	.005	1, 292	0.1	-.163**	-.148**	.023	1, 292	8.0**

*p = or <0.05; ** p = or <0.01; ***p = or <0.001.

r = Pearson product moment correlation coefficient; Beta = standardised regression coefficients.

The results of the theory-level analyses are shown in Table 5. The TPB explained 11.6% of the variance in behavioural simulation, SCT explained 12.1%, II explained 1.5%, and OLT explained 8.1%. In the cross-theoretical construct analysis, constructs from TPB (perceived behavioural control), II and CS-SRM (cause - ageing) were retained in the regression model, together explaining 16.5% of the variance in the scenario score (Table 4).

### Predicting behavioural intention

With the range of possible scores for intention of 1 to 7, the mean (SD) intention score was 2.1 (1.0); the median intention score was 1.6 with a range of 1 to 5.5. The constructs that predicted behavioural intention were: TPB (attitudes, subjective norms, perceived behavioural control); SCT (risk perception, outcome expectancy, self efficacy); OLT (anticipated consequences, evidence of habitual behaviour); CS-SRM (control - treatment, control - patient, control - doctor, cause - stress, emotional response, and coherence); knowledge; and PAP (Table 5).

The results of the theory level analyses are shown in Table 5. The TPB explained 25% of the variance in behavioural intention, SCT 21.5%, OLT 26.3%, CS-SRM 11.3%, knowledge 2.3%, and PAP explained 2.6%. In the cross-theoretical construct analysis, constructs from TPB (perceived behavioural control), OLT (evidence of habitual behaviour, outcome expectancy), CS-SRM (control - treatment) were retained in the regression model, together explaining 33.5% of the variance in intention (Table 4).

## Discussion

We have successfully developed and applied psychological theory-based questionnaires that have, in the context of ordering of lumbar spine x-rays in the management of patients with low back pain been able to predict two proxies for behaviour (behavioural simulation and intention) and (to a lesser extent) behaviour.

### Overall interpretation

Low back pain is a frequent presenting problem in primary care settings. However, the use of x-rays in clinical management of low back pain is relatively infrequent. In the theory level analysis predicting clinical behaviour, constructs relating to beliefs about consequences (SCT (risk perception) and CS-SRM (cause - poor prior medical treatment, cause - patient's own behaviour and cause-ageing, control - patient) and beliefs about capabilities (TPB (perceived behavioural control)) all significantly predicted behaviour. Looking across our two other outcome measures, there are also suggestions that beliefs about consequences (attitudes, outcome expectancies, risk perception, anticipated consequences) and beliefs about capabilities (PBC, self efficacy) may be important. In addition, II predicted behavioural simulation and OLT (evidence of habitual behaviour) predicted intention. The theories individually explained a significant proportion of the variance in behavioural simulation and intention, but overall were poorly predictive of behaviour. Together, these findings suggest both beliefs about consequences and beliefs about capabilities are likely determinants of lumbar spine x-ray requests.

This is a correlational study, so the causative aspects of the theories and their constructs remain untested in this population; but it is promising for the utility of applying psychological theory to changing clinical behaviour that the constructs are acting as the theories expect. These results suggest that an intervention that specifically targets predictive elements should have the greatest likelihood of success in influencing the implementation of this evidence-based practice.

The PRIME study has evaluated the predictive value of a range of theories across different behaviours (prescribing antibiotics for upper respiratory tract infections, or URTIs, taking dental radiographs, placing preventive fissure sealants), target professional groups (primary care doctors, dentists), and contexts [17,19,20,37]; we have demonstrated that different constructs predicted different proportions of the variance in the intention and behaviour. This raises the question of how best to identify relevant theories specific to different behaviours and clinical groups. One option would be to undertake preliminary work to identify the key construct domains that are likely to influence the target behaviours, and use them to specify potentially relevant theories [38,39].

### Strengths and weaknesses

Operationalising our behaviour of interest in the surveys that reflected the available behavioural data was challenging. Our behaviour of interest was managing patients with low back pain without referral for lumbar spine x-ray. However, we could only get behavioural data on the number of lumbar spine x-ray referrals ordered by primary care physicians. In general, we tried to word the survey questions to correspond to the available behavioural data (*e.g.*, 'when a patient presents with back pain, I have in mind to refer them for X-ray'). However, we found it difficult to frame some questions that corresponded to the behavioural data and clinically sensible. As a result the final questionnaire, included some questions worded in terms of doing the behaviour (*e.g.*, in general, referring patients with back pain for an X-ray would...) and some worded in terms of not doing the behaviour (*e.g.*, without an x-ray, how confident are you in your ability to...). This raises the issue of whether doing and not doing a behaviour are two sides of the same behaviour, or whether they represent linked but alternate behaviours. If the latter, the predictive ability of our survey instrument would be likely to be reduced.

Operationalising the constructs with theoretical fidelity was also challenging. A number of the models (OLT, II, CS-SRM) had not been operationalised in this way prior to the PRIME studies. OLT and II are usually used as intervention methods to change behaviour. However, both predicted behavioural simulation, and OLT predicted intention and behaviour. Since we undertook this study, some of the models have been adapted or enhanced, and different approaches to measurement have been developed -- for example, the post intentional action-coping planning enhancements of the TPB [40,41] and Verplanken's Self Reported Habit Index [42].

The CS-SRM pattern of results mirrored the overall picture of beliefs about consequences and capabilities being important. However, they did not predict behaviour, behavioural simulation, and intention particularly well. The model has previously been used mainly to refer to an individual's perceptions of their clinical condition; we used it to measure a clinician's perception of the condition in general. We had difficulty operationalising this model, and further work is needed to explore the utility of this theory to predict clinician behaviour.

There is a stepwise decrease in the proportion of variance across our dependent variables from intention to behavioural simulation (to behaviour) (Tables 2 and 4) as found in previous PRIME studies. Godin's review [16] of the predictive value of social cognitive models on professional behaviour showed a similar pattern, with social cognitive models explaining means of 13% of the variance in objectively measured behaviour (from 11 studies), 44% of self-reported behaviour (from four studies), and 59% of intention (from 72 studies). Our results are each lower than Godin's average figures, but all are within the range reported by other studies. However, our explanation of behaviour is at the very lowest limit of the reported range. In the previous PRIME studies, we have been able to explain 16% of the variance in general dental practitioners' use of dental radiographs [37] and 6% of primary care physicians prescribing of antibiotics for patients who present with an URTI [20]. This suggests that our operationalisation of the models was likely to have been good, and raises the question of why the models did not work as well for ordering lumbar spine x-rays by primary care physicians.

We can identify three potential explanations. Firstly, there was poor correspondence between the behaviour specified in the survey and the measured behaviour as mentioned above. This highlights the importance of clear and consistent framing of the questions and concordance with the measured behaviour. In the previous PRIME papers, the behaviours specified in the surveys and the measured behaviours were: dental radiographs (survey - use of intra-oral radiographs in patient management, data - the number of intra oral radiographs taken per course of treatment (good concordance)) [18], and antibiotics (survey - prescribing an antibiotic for patients presenting with URTIs, managing patients without an antibiotic, data - number of likely URTI relevant antibiotic prescriptions per 100 patients registered (weak concordance)) [20].

Second, there was potentially excess observational error (noise) in our behaviour measure. X-ray-ordering data was chosen because it was available from routine data sources, and was therefore inexpensive to collect. Low back pain was chosen because it was more likely that a request for an investigation would be attributed to the primary care doctor who issued it. Despite this, anecdotally we believe that there may be errors in the attribution of x-rays to doctors, with radiology departments reporting that requests could be reported to the correct practice but attributed to the wrong primary care doctor. In addition, we attempted to standardise our behaviour by the number of patients registered with the primary care doctor to reflect differences in workloads of the participating primary care doctors. We only had data on the total number of patients and number of primary care doctors in each practice, and so calculated an average list size per primary care doctor within each practice. This is a relatively crude standardisation approach that does not take account of likely variations of workload within practices (not all primary care doctors in the same practice will have the same workload) and variations in presentation of the target condition (not all primary care doctors will have same rate of presentation of low back pain). In the previous PRIME studies, these issues were likely to have been more problematic in the antibiotic study rather than the fissure sealant study (where data were abstracted from a claims database). These issues reflect some of the challenges of using routine data to measure behaviour relating to the level of clinical detail available (we could not estimate the number of patients each primary care doctor saw presenting with back pain) and problems of attribution of clinical actions to specific primary care doctors. In future studies of this kind, it will be important to invest more in the measurement of the behavioural data. These issues are likely to be less problematic in population-based large administrative database facilities where there may be detailed understanding of the content of the available data and their limitations. Alternatively it could be possible to collect behavioural data directly.

Thirdly, we used a different analytical approach to analyse the behavioural data. Previous PRIME studies have used multiple regression analyses and used the adjusted R^2 ^statistic from ordinary least squares (OLS) regression to quantify the proportion of variance explained by the models. In the current study, when we conducted multiple regression analyses of behavioural simulation and intention, we observed similar magnitude R^2 ^statistics for behavioural simulation and intention models. However given the distribution of the lumbar spine x-ray data, we had to use negative binomial regression for the behavioural analysis. A direct equivalent to the adjusted R^2 ^statistic does not exist for negative binomial regression. There are several pseudo R^2 ^statistics that mimic OLS R^2 ^in the sense that they can range over the scale 0 to 1 with higher values indicating a better fit of models to data. We present the results for various goodness to fit models that suggest that, in general, the resulting models overall were poorly predictive of the behavioural data. However these pseudo R^2 ^values cannot be used to compare the performance of competing theoretical models across different data sets, making comparisons of proportion variation explained with previous PRIME study surveys qualitative only. To explore the likely comparability of these results with previous PRIME studies, we undertook an OLS regression of square root transformed behavioural data and observed an R^2 ^statistic of 0.05, which is at the lower end of the observed R^2 ^statistics from previous PRIME studies. Together, we believe these data suggest that the models may be performing similarly to those in previous PRIME studies and the analytical approach required due to the negative binomial distribution is obscuring this.

Our final response rate was not high compared to what would be expected for a postal questionnaire survey to healthcare professionals. Following the report by Cummings *et al*. that up to 1995, response rates of surveys of healthcare professionals remained constant at approximately 60% [43], Cook *et al*. demonstrated that by 2005 response rates in surveys of healthcare professionals had slightly declined to an average of 57.5% [44]. Given this, we cannot exclude the possibility of selection bias in respondents and should be cautious about generalising from our respondents to the population of UK primary care physicians. However, this may be less of an issue at this exploratory stage of using these methods, as the purpose of the study was theory testing and an exploration of the predictive ability of theories to explain variations in behaviour. Our aim was not to generate data that was representative, but to receive our pre-specified number of responses from a population who had a range of behaviour, reported a range of behavioural simulation and intention, and who reported a range of cognitions. The study achieved this aim.

## Conclusions

This study provides evidence that psychological models may be useful in understanding and predicting clinical behaviour. Taking a theory-based approach enables the creation of a replicable methodology for identifying factors that predict clinical behaviour. However, there remain conceptual challenges in operationalising a number of the models and a range of methodological challenges in terms of instrument development and measurement of behaviour that have to be surmounted before these methods could be regarded as routine.

## Competing interests

Martin Eccles is Co-Editor in Chief of *Implementation Science*; Jeremy Grimshaw is a member of the editorial board of *Implementation Science*. All editorial decisions on this article were made by Robbie Foy, Deputy Editor.

## Authors' contributions

AW, MPE, JG, MJ, and NP conceived the study. MJ, LS, GM, RT, DB, and MPE contributed to the daily running of the study. MJ and NS oversaw the analysis, which was conducted by GM. All authors commented on sequential drafts of the paper and agreed the final draft.

## Supplementary Material

Additional File 1 PRIME Lumbar Spine Survey Instrument Click here for file

Additional File 2 Goodness of fit models for the negative binomial regression analysis Click here for file

## References

[B1] Seddon ME Marshall MN Campbell SM Roland MO Systematic review of studies of quality of clinical care in general practice in the UK, Australia and New Zealand Qual Health Care 2001 10 152 158 10.1136/qhc.0100152 11533422 PMC1743427

[B2] Grol R Successes and failures in the implementation of evidence-based guidelines for clinical practice Med Care 2001 39 II46 II54 11583121 10.1097/00005650-200108002-00003

[B3] McGlynn EA Asch SM Adams J Keesey J Hicks J DeCristofaro A The quality of health care delivered to adults in the United States N Engl J Med 2003 348 2635 2645 10.1056/NEJMsa022615 12826639

[B4] Jarvik JG Deyo RA Diagnostic evaluation of low back pain with emphasis on imaging Ann Intern Med 2002 137 586 597 12353946 10.7326/0003-4819-137-7-200210010-00010

[B5] Royal College of Radiologists Making the best use of a Department of Radiology 1998 London: Royal College of Radiologists

[B6] Australian Acute Musculoskeletal Pain Guidelines Group (AAMPGG) Evidence-based management of acute musculoskeleta pain 2003 Brisbane: Australian Academic Press

[B7] Savigny P Kuntze S Watson P Underwood M Ritchie G Cotterell M Low back pain: early management of persistent non-specific low back pain 2009 London: National Collaborating Centre for Primary Care and Royal College of General Practitioners 20704057

[B8] National Institute for Health and Clinical Excellence NICE clinical guideline 88 Low back pain early management of persistent non-specific low back oain 2009 London: National Institute for Health and Clinical Excellence

[B9] Matowe L Ramsay CR Grimshaw JM Gilbert FJ Macleod MJ Needham G Effects of mailed dissemination of the Royal College of Radiologists' guidelines on general practitioner referrals for radiography: a time series analysis Clin Radiol 2002 57 575 578 10.1053/crad.2001.0894 12096854

[B10] Eccles M Steen N Grimshaw J Thomas L McNamee P Soutter J Effect of audit and feedback, and reminder messages on primary-care radiology referrals: a randomised trial Lancet 2001 357 1406 1409 10.1016/S0140-6736(00)04564-5 11356439

[B11] Grimshaw JM Shirran L Thomas R Mowatt G Fraser C Bero L Changing provider behavior: an overview of systematic reviews of interventions Med Care 2001 39 II2 45 11583120

[B12] Grimshaw JM Thomas RE Maclennan G Fraser C Ramsay CR Vale L Effectiveness and efficiency of guideline dissemination and implementation strategies Health Technol Assess 2004 8 iii 72 14960256 10.3310/hta8060

[B13] Foy R Eccles MP Jamtvedt G Young J Grimshaw JM Baker R What do we know about how to do audit and feedback? Pitfalls in applying evidence from a systematic review BMC Health Serv Res 2005 5 50 10.1186/1472-6963-5-50 16011811 PMC1183206

[B14] Eccles M Grimshaw J Walker A Johnston M Pitts N Changing the behavior of healthcare professionals: the use of theory in promoting the uptake of research findings J Clin Epidemiol 2005 58 107 112 10.1016/j.jclinepi.2004.09.002 15680740

[B15] The Improved Clinical Effectiveness through Behavioural Research Group (ICEBeRG) Designing theoretically-informed implementation interventions Implement Sci 2006 1 4 16722571 10.1186/1748-5908-1-4 PMC1436012

[B16] Godin G Belanger-Gravel A Eccles M Grimshaw J Healthcare professionals' intentions and behaviours: A systematic review of studies based on social cognitive theories Implement Sci 2008 3 36 10.1186/1748-5908-3-36 18631386 PMC2507717

[B17] Walker AE Grimshaw J Johnston M Pitts N Steen N Eccles M PRIME--PRocess modelling in ImpleMEntation research: selecting a theoretical basis for interventions to change clinical practice BMC Health Serv Res 2003 3 22 10.1186/1472-6963-3-22 14683530 PMC317340

[B18] Bonetti D Pitts NB Eccles M Grimshaw J Johnston M Steen N Applying psychological theory to evidence-based clinical practice: identifying factors predictive of taking intra-oral radiographs Soc Sci Med 2006 63 1889 1899 10.1016/j.socscimed.2006.04.005 16843579

[B19] Bonetti D Johnston M Clarkson JE Grimshaw J Pitts NB Eccles M Applying psychological theories to evidence-based clinical practice: identifying factors predictive of placing preventive fissure sealants Implement Sci 2010 5 25 10.1186/1748-5908-5-25 20377849 PMC2864198

[B20] Eccles MP Grimshaw JM Johnston M Steen N Pitts NB Thomas R Applying psychological theories to evidence-based clinical practice: Identifying factors predictive of managing upper respiratory tract infections without antibiotics Implement Sci 2007 2 26 10.1186/1748-5908-2-26 17683558 PMC2042498

[B21] Ajzen I The theory of planned behavior Organizational Behavior and Human Decision Processes 1991 50 179 211 10.1016/0749-5978(91)90020-T

[B22] Bandura A Self-efficacy: toward a unifying theory of behavioral change Psychol Rev 1977 84 191 215 847061 10.1037//0033-295x.84.2.191

[B23] Blackman D Operant conditioning an experimental analysis of behaviour 1974 London: Methuen

[B24] Gollwitzer PM Stroebe W, Hewstone M Goal achievement: the role of intentions European Review of Social Psychology 1993 Chichester: Wiley

[B25] Leventhal H Nerenz D Steele DJ Handbook of psychology and health 1984 New Jersey: Lawrence Erlbaum

[B26] Weinstein ND The precaution adoption process Health Psychol 1988 7 355 386 3049068 10.1037//0278-6133.7.4.355

[B27] Weinstein ND Rothman A Sutton SR Stage theories of health behaviour: conceptual and methodological issues Health Psychol 1998 17 290 299 9619480 10.1037//0278-6133.17.3.290

[B28] Walker AE Grimshaw J Johnston M Pitts N Steen N Eccles M PRIME--PRocess modelling in ImpleMEntation research: selecting a theoretical basis for interventions to change clinical practice BMC Health Serv Res 2003 3 22 10.1186/1472-6963-3-22 14683530 PMC317340

[B29] Conner M Sparks P Conner M, Norman P The theory of planned behaviour and health behaviours Predicting health behaviour Research and practice with social cognition models 1996 1 Buckingham: Open University Press 121 162

[B30] Francis JJ Eccles M Johnston M Walker A Grimshaw J Foy R Constructing questionnaires based on the theory of planned behaviour A manual for health services researchers 2004

[B31] Bandura A Self-efficacy the exercise of control 1997 New York: Freeman

[B32] Bandura A Norman P, Abraham C, Conner M Health promotion from the persepctive of social cognitive theory Understanding and changing Health Behaviour from Health Beliefs to Self-Regulation 2000 Amsterdam: Harwood

[B33] Moss-Morris R Weinman J Petrie KJ Horne R Cameron LD Buick D The revised illness perception questionnaire (IPQ-R) Psychol Health 2002 17 1 16 10.1080/08870440290001494

[B34] Hrisos S Eccles MP Francis JJ Dickinson HO Kaner EF Beyer F Are there valid proxy measures of clinical behaviour? A systematic review Implement Sci 2009 4 37 10.1186/1748-5908-4-37 19575790 PMC2713194

[B35] Green SB How many subjects does it take to do a regression analysis? Multivariate Behavioural Research 1991 26 499 510 10.1207/s15327906mbr2603_7 26776715

[B36] Freese J Long JS Regression models for categorical dependent variables using Stata 2006 College Station: Stata Press

[B37] Bonetti D Pitts NB Eccles M Grimshaw J Steen N Glidewell L Applying psychological theory to evidence-based clinical practice: identifying factors predictive of taking intra-oral radiographs Soc Sci Med 2006 63 1889 1899 10.1016/j.socscimed.2006.04.005 16843579

[B38] Francis JJ Tinmouth A Stanworth SJ Grimshaw JM Johnston M Hyde C Using theories of behaviour to understand transfusion prescribing in three clinical contexts in two countries: development work for an implementation trial Implement Sci 2009 4 70 10.1186/1748-5908-4-70 19852832 PMC2777847

[B39] Francis JJ Stockton C Eccles MP Johnston M Cuthbertson BH Grimshaw JM Evidence-based selection of theories for designing behaviour change interventions: using methods based on theoretical construct domains to understand clinicians' blood transfusion behaviour Br J Health Psychol 2009 14 625 646 10.1348/135910708X397025 19159506

[B40] Sniehotta FF Scholz U Schwarzer R Action plans and coping plans for physical exercise: A longitudinal intervention study in cardiac rehabilitation Br J Health Psychol 2006 11 23 37 10.1348/135910705X43804 16480553

[B41] Sniehotta FF Schwarzer R Scholz U Schuz B Action planning and coping planning for long term lifestyle change: theory and assessment European Journal of Social Psychology 2005 35 565 576 10.1002/ejsp.258

[B42] Verplanken B Orbell S Reflections on past behavior: a self-report index of habit strength Journal of Applied Social Psychology 2003 33 1313 1330 10.1111/j.1559-1816.2003.tb01951.x

[B43] Cummings SM Savitz LA Konrad TR Reported response rates to mailed physician questionnaires Health Serv Res 2001 35 1347 1355 11221823 PMC1089194

[B44] Cook JV Dickinson HO Eccles MP Response rates in postal surveys of healthcare professionals between 1996 and 2005: an observational study BMC Health Serv Res 2009 9 160 10.1186/1472-6963-9-160 19751504 PMC2758861

