# Supplementary material for: Applying psychological theories to evidence-based clinical practice: identifying factors predictive of lumbar spine x-ray for low back pain in UK primary care practice
Source: Implement Sci. 2011 May 28;6:55. doi: 10.1186/1748-5908-6-55 (PMC3125229; doi:10.1186/1748-5908-6-55)
Supplement: Additional File 1 — PRIME Lumbar Spine Survey Instrument [file 1748-5908-6-55-S1.DOC]

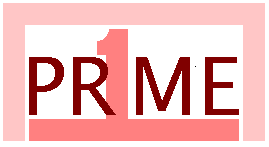


**MANAGEMENT OF PATIENTS WITH LOW BACK PAIN IN PRIMARY CARE**


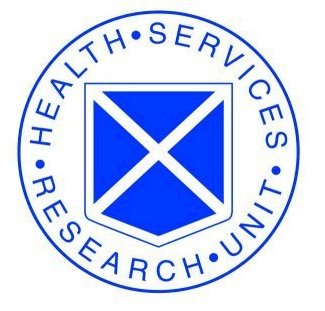

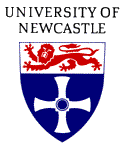


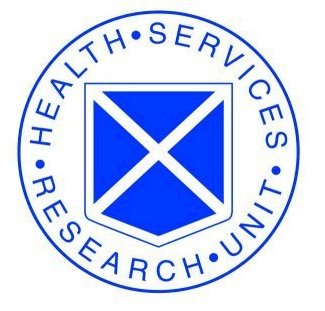


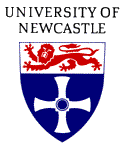


First of all, thank you for participating in this research. This questionnaire concerns factors that may influence your decision to refer a patient with back pain for a lumbo-sacral spine X-ray, in addition to clinical factors. The questions are based on a previous study of GP attitudes toward X-rays. You will notice that some of the questions *seem* to be very alike. They are different and it is important to answer them all. Try **not** to make a deliberate effort to be consistent - it is because the different formats can mean different things to people that we pose the questions in these ways. The main thing to remember is to answer as directly as possible, not what you think you should answer or what we want to see. Your answers are completely confidential. Try not to take too long over each response - what first comes to mind is more likely reflect what you really believe.

***Background***

**Please check that your contact details are correct and amend if necessary**.

| **Your Contact Phone Number** |  | **e-mail** |  |
| --- | --- | --- | --- |

| B1. Male |  | Female |  |  |
| --- | --- | --- | --- | --- |

| B2. How long have you been qualified as a doctor? |  |  |  | **Years** |
| --- | --- | --- | --- | --- |

| B3. How many full time equivalent principals (excluding you) are there in your |  |  |  |
| --- | --- | --- | --- |
| practice? |  |  |  |

| B4. Are you a GP trainer in a vocational training scheme? | Yes |  | No |  |  |
| --- | --- | --- | --- | --- | --- |

| B5. How many clinical sessions (0.5 days) do you work per week on average? |  |  |  |
| --- | --- | --- | --- |

| B6. What is your approximate total practice list size? |  |  |  |  |  |  |  |
| --- | --- | --- | --- | --- | --- | --- | --- |

***This page will be removed and stored separately from the remainder of the questionnaire to preserve confidentiality***

**This questionnaire refers to patients presenting with back pain in general practice and to lumbo-sacral (lumbar) spine X-rays. Please treat all sub-questions e.g. 1a) 1b) 1c), as separate questions and answer each one*.***

| Section 1: Referring patients |
| --- |

| **1.** | a) | **From memory, approximately how many of the last 10 patients who presented with back pain for the *first* time, did you refer for an X-ray:** |
| --- | --- | --- |

| 0 | 1 | 2 | 3 | 4 | 5 | 6 | 7 | 8 | 9 | 10 |
| --- | --- | --- | --- | --- | --- | --- | --- | --- | --- | --- |

|  | b) | **From memory, approximately how many of the last 10 patients who presented with back pain for the**  ***second* time, did you refer for an X-ray:** |
| --- | --- | --- |

| 0 | 1 | 2 | 3 | 4 | 5 | 6 | 7 | 8 | 9 | 10 |
| --- | --- | --- | --- | --- | --- | --- | --- | --- | --- | --- |

Strongly disagree Strongly agree

| **2.** | **I feel under pressure from my patients *to* refer for an X-ray** | 1 | 2 | 3 | 4 | 5 | 6 | 7 |
| --- | --- | --- | --- | --- | --- | --- | --- | --- |
| **3.** | **I feel under pressure from physiotherapists *to* refer patients for an X-ray** | 1 | 2 | 3 | 4 | 5 | 6 | 7 |
| **4.** | **I feel under pressure from the NHS *not to* refer patients for an X-ray** | 1 | 2 | 3 | 4 | 5 | 6 | 7 |
| **5.** | **I feel under pressure from colleagues *not to* refer patients for an X-ray** | 1 | 2 | 3 | 4 | 5 | 6 | 7 |
| **6.** | **If I refer a patient with back pain for an X-ray, then I will think of myself as a caring GP** | 1 | 2 | 3 | 4 | 5 | 6 | 7 |
| **7.** | **If I refer a patient with back pain for an X-ray, then I will think of myself as a competent GP** | 1 | 2 | 3 | 4 | 5 | 6 | 7 |
| **8.** | **It is highly likely that patients with back pain will be worse off if I do not refer them for an X-ray** | 1 | 2 | 3 | 4 | 5 | 6 | 7 |
| **9.** | In general, referring patients with back pain **for an X-ray** **would:** | Strongly disagree Strongly agree | | | | | | |
|  | a) Reassure them | 1 | 2 | 3 | 4 | 5 | 6 | 7 |
|  | b) Reduce my own uncertainty | 1 | 2 | 3 | 4 | 5 | 6 | 7 |
|  | c) Increase my confidence in managing their symptoms | 1 | 2 | 3 | 4 | 5 | 6 | 7 |
|  | d) Mean less time spent in future with that patient in regard to their back pain | 1 | 2 | 3 | 4 | 5 | 6 | 7 |

| 10. | | If I start routinely referring patients with back pain Strongly disagree Strongly agreefor an X-ray then: | | | | | | | | |
| --- | --- | --- | --- | --- | --- | --- | --- | --- | --- | --- |
|  | a) | | On balance, my life as a GP will be easier in the long run | 1 | 2 | 3 | 4 | 5 | 6 | 7 |
|  | b) | | On balance, the consequences for me as a GP (e.g. stress, time, future requirements etc.) will be worse | 1 | 2 | 3 | 4 | 5 | 6 | 7 |

| 11. | Not at all ExtremelyHow confident are you in your ability to: confident confident |
| --- | --- |

| a) | Diagnose back problems without using an X-ray report? | 1 | 2 | 3 | 4 | 5 | 6 | 7 |
| --- | --- | --- | --- | --- | --- | --- | --- | --- |
| b) | Treat back problems without using an X-ray report? | 1 | 2 | 3 | 4 | 5 | 6 | 7 |
| c) | Present a diagnosis to a problematic patient without an X-ray report? | 1 | 2 | 3 | 4 | 5 | 6 | 7 |
| d) | Present a diagnosis to an anxious patient without an X-ray report? | 1 | 2 | 3 | 4 | 5 | 6 | 7 |

Strongly disagree Strongly agree

| **12...** | a) | When I see a patient with back pain, I automatically consider referring them for an X-ray | 1 | 2 | 3 | 4 | 5 | 6 | 7 |
| --- | --- | --- | --- | --- | --- | --- | --- | --- | --- |
|  | b) | It is my usual practice to refer patients with back pain for an X-ray | 1 | 2 | 3 | 4 | 5 | 6 | 7 |
|  | c) | I aim to refer patients with back pain for an X-ray as part of patient management | 1 | 2 | 3 | 4 | 5 | 6 | 7 |

| **13.** | **Given 10 patients presenting for the *second* time with the same episode of back pain, how many patients would you expect to refer for a lumbar X-ray?** |
| --- | --- |

| 0 | 1 | 2 | 3 | 4 | 5 | 6 | 7 | 8 | 9 | 10 |
| --- | --- | --- | --- | --- | --- | --- | --- | --- | --- | --- |

| 14. | Which of these sentences *most* characterises you at the moment (Please tick *only one* of the 7 boxes) | | | |  |
| --- | --- | --- | --- | --- | --- |
|  |  | 1 | I have not yet thought about changing the number of lumbar X-rays I currently request |  | |
|  |  | 2 | It has been a while since I have thought about changing the number of lumbar X-rays I request |  | |
|  |  | 3 | I have thought about it and decided that I *will not change* the number of lumbar X-rays I request |  | |
|  |  | 4 | I have decided that I *will request more* lumbar X-rays |  | |
|  |  | 5 | I have decided that I *will request less* lumbar X-rays |  | |
|  |  | 6 | I have *already* done something about increasing the number of lumbar X-rays I request |  | |
|  |  | 7 | I have *already* done something about decreasing the number of lumbar X-rays I request |  | |

| **15.** | a) | | Think about the last time you referred a patient for a lumbar spine X-ray and felt pleased that you had done so. Do you think the result of this episode has made you: | | | | | | | | | | |
| --- | --- | --- | --- | --- | --- | --- | --- | --- | --- | --- | --- | --- | --- |
|  | | More likely to refer | |  | **Less likely**  **to refer** |  | **Unchanged** |  | **Not sure** |  | **Never occurred** |  |  |

|  | b) | Think about the last time you referred a patient for a lumbar spine X-ray and felt sorry that you had done so. Do you think the result of this episode has made you: | | | | | | | | | |
| --- | --- | --- | --- | --- | --- | --- | --- | --- | --- | --- | --- |
|  | More likely  to refer | |  | **Less likely**  **to refer** |  | **Unchanged** |  | **Not sure** |  | **Never occurred** |  |

|  | c) | Think about the last time you decided not to refer a patient for a lumbar spine X-ray and felt sorry that you had not done so. Do you think the result of this episode has made you: | | | | | | | | | |
| --- | --- | --- | --- | --- | --- | --- | --- | --- | --- | --- | --- |
|  | More likely  to refer | |  | **Less likely**  **to refer** |  | **Unchanged** |  | **Not sure** |  | **Never occurred** |  |

|  | d) | Think about the last time you decided not to refer a patient for a lumbar spine X-ray and felt pleased that you had not done so. Do you think the result of this episode made you: | | | | | | | | | |
| --- | --- | --- | --- | --- | --- | --- | --- | --- | --- | --- | --- |
|  | More likely  to refer | |  | **Less likely**  **to refer** |  | **Unchanged** |  | **Not sure** |  | **Never occurred** |  |

| Section 2: managing patients with back pain |
| --- |

| 1. | Without an X-ray, I find it difficult to manage patients Strongly disagree Strongly agree **with back pain who:** |
| --- | --- |

###

| a) | Visit the surgery a lot because of their back pain | 1 | 2 | 3 | 4 | 5 | 6 | 7 |
| --- | --- | --- | --- | --- | --- | --- | --- | --- |
| b) | Are frequent attenders in general | 1 | 2 | 3 | 4 | 5 | 6 | 7 |
| c) | Do not often visit the surgery | 1 | 2 | 3 | 4 | 5 | 6 | 7 |
| d) | Are taking a lot of analgesics for back pain | 1 | 2 | 3 | 4 | 5 | 6 | 7 |
| e) | Expect me to refer them for an X-ray because of their back pain | 1 | 2 | 3 | 4 | 5 | 6 | 7 |
| f) | Do not have a past history of back pain | 1 | 2 | 3 | 4 | 5 | 6 | 7 |
| g) | Have a past history of back pain | 1 | 2 | 3 | 4 | 5 | 6 | 7 |
| h) | Really want me to refer them for an X-ray | 1 | 2 | 3 | 4 | 5 | 6 | 7 |
| i) | Are very emotionally upset | 1 | 2 | 3 | 4 | 5 | 6 | 7 |
| j) | Worry a great deal about their back pain | 1 | 2 | 3 | 4 | 5 | 6 | 7 |

| 2. | Generally I find it difficult to: Strongly disagree Strongly agree | | | | | | | |  |
| --- | --- | --- | --- | --- | --- | --- | --- | --- | --- |
| a) | Diagnose back problems without an X-ray report | 1 | 2 | 3 | 4 | 5 | 6 | 7 | |
| b) | Treat back problems without an X-ray report | 1 | 2 | 3 | 4 | 5 | 6 | 7 | |
| c) | Present a diagnosis to a problematic patient without an X-ray report | 1 | 2 | 3 | 4 | 5 | 6 | 7 | |
| d) | Present a diagnosis to an anxious patient without an X-ray report | 1 | 2 | 3 | 4 | 5 | 6 | 7 | |
| **3.**  a) | I would like to avoid referring patients for a lumbar X-ray, but I don’t really know if I can | 1 | 2 | 3 | 4 | 5 | 6 | 7 | |
| b) | Whether I refer patients for a lumbar X-ray is entirely up to me | 1 | 2 | 3 | 4 | 5 | 6 | 7 | |
| c) | I am confident that I can avoid referring patients for a lumbar X-ray if I want to | 1 | 2 | 3 | 4 | 5 | 6 | 7 | |
| d) | I can overcome all obstacles, whatever they may be, in managing back pain without an X-ray report | 1 | 2 | 3 | 4 | 5 | 6 | 7 | |

| **4.** | **In general:** | Strongly disagree Strongly agree | | | | | | | |
| --- | --- | --- | --- | --- | --- | --- | --- | --- | --- |
| a) | The possible harm to the patient of a lumbar spine X-ray is outweighed by its benefits | | 1 | 2 | 3 | 4 | 5 | 6 | 7 |
| b) | Referring patients for a lumbar spine X-ray is more often bad practice than good practice | | 1 | 2 | 3 | 4 | 5 | 6 | 7 |

Unimportant Important

| **5.** | **Reassuring patients with back pain is** | 1 | 2 | 3 | 4 | 5 | 6 | 7 |
| --- | --- | --- | --- | --- | --- | --- | --- | --- |
| **6.** | **Reducing my own uncertainty about patients with back pain is** | 1 | 2 | 3 | 4 | 5 | 6 | 7 |
| **7.** | **Being confident about managing patient’s with back pain is** | 1 | 2 | 3 | 4 | 5 | 6 | 7 |
| **8.** | **Spending less time in future with patients for their back pain is** | 1 | 2 | 3 | 4 | 5 | 6 | 7 |
| **9.** | **Thinking of myself as a caring GP is** | 1 | 2 | 3 | 4 | 5 | 6 | 7 |
| **10.** | **Thinking of myself as a competent GP is** | 1 | 2 | 3 | 4 | 5 | 6 | 7 |

| 11. | How motivated are you to do: Not at all Very much | | | | | | | | |
| --- | --- | --- | --- | --- | --- | --- | --- | --- | --- |
|  | a) | What patients think you should | 1 | 2 | 3 | 4 | 5 | 6 | 7 |
|  | b) | What physiotherapists think you should | 1 | 2 | 3 | 4 | 5 | 6 | 7 |
|  | c) | What the NHS thinks you should | 1 | 2 | 3 | 4 | 5 | 6 | 7 |
|  | d) | What colleagues think you should | 1 | 2 | 3 | 4 | 5 | 6 | 7 |

| 12. | Without an X-ray, how confident are you in your ability Not at all Extremelyto treat patients with back pain who: confident confident | | | | | | | | |
| --- | --- | --- | --- | --- | --- | --- | --- | --- | --- |
|  | a) | Visit the surgery a lot because of their back pain | 1 | 2 | 3 | 4 | 5 | 6 | 7 |
|  | b) | Are frequent attenders in general | 1 | 2 | 3 | 4 | 5 | 6 | 7 |
|  | c) | Do not often visit the surgery | 1 | 2 | 3 | 4 | 5 | 6 | 7 |
|  | d) | Are taking a lot of analgesics for their back pain | 1 | 2 | 3 | 4 | 5 | 6 | 7 |
|  | e) | Expect me to refer them for an X-ray | 1 | 2 | 3 | 4 | 5 | 6 | 7 |
|  | f) | Do not have a past history of back pain | 1 | 2 | 3 | 4 | 5 | 6 | 7 |
|  | g) | Have a past history of back pain | 1 | 2 | 3 | 4 | 5 | 6 | 7 |
|  | h) | Really want me to refer them for an X-ray | 1 | 2 | 3 | 4 | 5 | 6 | 7 |
|  | i) | Are very emotionally upset | 1 | 2 | 3 | 4 | 5 | 6 | 7 |
|  | j) | Worry a great deal about their back pain | 1 | 2 | 3 | 4 | 5 | 6 | 7 |

Strongly disagree Strongly agree

| **13.** | **When a patient presents with back pain, I have in mind to refer them for X-ray** | 1 | 2 | 3 | 4 | 5 | 6 | 7 |
| --- | --- | --- | --- | --- | --- | --- | --- | --- |
| **14.** | **I intend to refer patients with back pain for an X-ray as part of their management** | 1 | 2 | 3 | 4 | 5 | 6 | 7 |
| 15. | **Currently, my standard method of managing patients with back pain does not include referring them for an X-ray** | 1 | 2 | 3 | 4 | 5 | 6 | 7 |

| 16. | | | a) | | Do you have a clear idea how you would want to manage patients with back pain? | | | | | | | | | |  |
| --- | --- | --- | --- | --- | --- | --- | --- | --- | --- | --- | --- | --- | --- | --- | --- |
|  | |  | |  | |  |  |  |  | |  |  |  | | |
|  | |  | |  | | Yes |  | No |  | | Unsure |  |  | | |
|  | |  | |  | |  |  |  |  | |  |  |  | | |
|  | b) If Yes, please describe it briefly | | | | | | | | | | | | |  | |
|  |  | | | | | | | | |  | | | |  | |
|  |  | | | | | | | | |  | | | |  | |
|  |  | | | | | | | | |  | | | |  | |
|  |  | | | | | | | | |  | | | |  | |

| Section 3: Back pain |
| --- |

| 1. | **Back pain as seen in general practice:**  ***Strongly disagree*** ***Strongly agree*** | | | | | | | | |
| --- | --- | --- | --- | --- | --- | --- | --- | --- | --- |
|  | a) | Is generally of an intense nature | 1 | 2 | 3 | 4 | 5 | 6 | 7 |
|  | b) | Has many symptoms | 1 | 2 | 3 | 4 | 5 | 6 | 7 |
|  | c) | Should concern doctors | 1 | 2 | 3 | 4 | 5 | 6 | 7 |
|  | d) | Is likely to be permanent rather than temporary | 1 | 2 | 3 | 4 | 5 | 6 | 7 |
|  | e) | Is likely to be recurrent rather than a one off episode | 1 | 2 | 3 | 4 | 5 | 6 | 7 |
|  | f) | Will pass quickly | 1 | 2 | 3 | 4 | 5 | 6 | 7 |
|  | g) | Is very unpredictable | 1 | 2 | 3 | 4 | 5 | 6 | 7 |
|  | h) | Changes a great deal from day to day | 1 | 2 | 3 | 4 | 5 | 6 | 7 |

|  | 2. | **Back pain is caused by:**  ***Strongly disagree*** ***Strongly agree*** | | | | | | | | | | | | | | | | | |
| --- | --- | --- | --- | --- | --- | --- | --- | --- | --- | --- | --- | --- | --- | --- | --- | --- | --- | --- | --- |
|  |  | a) | | | Stress or worry | 1 | | 2 | | 3 | | 4 | | 5 | | 6 | | 7 | |
|  |  | b) | | | Family problems | 1 | | 2 | | 3 | | 4 | | 5 | | 6 | | 7 | |
|  |  | c) | | | Poor medical care in the past | 1 | | 2 | | 3 | | 4 | | 5 | | 6 | | 7 | |
|  |  | d) | | | The patient’s own behaviour | 1 | | 2 | | 3 | | 4 | | 5 | | 6 | | 7 | |
|  |  | e) | | | Ageing | 1 | | 2 | | 3 | | 4 | | 5 | | 6 | | 7 | |
|  |  | f) | | | Chance or bad luck | 1 | | 2 | | 3 | | 4 | | 5 | | 6 | | 7 | |
|  |  | g) | | | Overwork | 1 | | 2 | | 3 | | 4 | | 5 | | 6 | | 7 | |
|  |  | h) | | | Stress or worry | 1 | | 2 | | 3 | | 4 | | 5 | | 6 | | 7 | |
|  | | | | ***Strongly disagree Strongly agree*** | | | | | | | | | | | | | | | |
| **3.** | | | **There is very little that can be done to improve back pain** | | | | 1 | | 2 | | 3 | | 4 | | 5 | | 6 | | 7 |
| **4.** | | | **There is nothing which can help back pain** | | | | 1 | | 2 | | 3 | | 4 | | 5 | | 6 | | 7 |
| **5.** | | | **Treatment can control back pain** | | | | 1 | | 2 | | 3 | | 4 | | 5 | | 6 | | 7 |
| **6.** | | | **There is a lot which the patient can do to control the symptoms of back pain** | | | | 1 | | 2 | | 3 | | 4 | | 5 | | 6 | | 7 |
| **7.** | | | **What the patient does can determine whether back pain gets better or worse** | | | | 1 | | 2 | | 3 | | 4 | | 5 | | 6 | | 7 |
| **8.** | | | **Nothing I do will affect the patient’s back pain** | | | | 1 | | 2 | | 3 | | 4 | | 5 | | 6 | | 7 |
| **9.** | | | **What I do can determine whether the patient’s back pain gets better or worse** | | | | 1 | | 2 | | 3 | | 4 | | 5 | | 6 | | 7 |
| **10.** | | | In general, back pain is a serious condition | | | | 1 | | 2 | | 3 | | 4 | | 5 | | 6 | | 7 |

|  | ***Strongly disagree Strongly agree*** | | | | | | | | |
| --- | --- | --- | --- | --- | --- | --- | --- | --- | --- |
| **11.** | | **Back pain does not have much effect on the patient’s life** | 1 | 2 | 3 | 4 | 5 | 6 | 7 |
| **12.** | | **Back pain has serious financial consequences** | 1 | 2 | 3 | 4 | 5 | 6 | 7 |
| **13.** | | **I have a clear picture or understanding of back pain** | 1 | 2 | 3 | 4 | 5 | 6 | 7 |
| **14.** | | The symptoms of back pain are puzzling to me | 1 | 2 | 3 | 4 | 5 | 6 | 7 |
| **15.** | | **I get depressed when I think about back pain** | 1 | 2 | 3 | 4 | 5 | 6 | 7 |
| **16.** | | **Seeing patients with back pain does not worry me** | 1 | 2 | 3 | 4 | 5 | 6 | 7 |
| **17.** | | **Seeing patients with back pain makes me feel angry** | 1 | 2 | 3 | 4 | 5 | 6 | 7 |
| **18.** | | **Seeing patients with back pain can affect me emotionally**  **(e.g. can make me feel helpless, anxious, or distressed)** | 1 | 2 | 3 | 4 | 5 | 6 | 7 |

| Section 4: use of lumbar spine x-rays in managing patients with back pain |
| --- |

|  |  | | ***True*** | ***False*** | ***Not sure*** |
| --- | --- | --- | --- | --- | --- |
| **1.** | a) | The presence of spondolytic changes **on a lumbar spine X-ray** correlates well with back pain | 1 | 2 | 3 |
|  | b) | **A lumbar spine X-ray** involves a large dose of radiation, of the order of the equivalent of 60 – 100 chest X-rays | 1 | 2 | 3 |
|  | c) | **A lumbar spine X-ray** is a sensitive and specific investigation for diagnosing spinal bony secondaries from prostate cancer | 1 | 2 | 3 |
|  | d) | **A lumbar spine X-ray** can be useful in the investigation of persisting back pain in adolescents | 1 | 2 | 3 |
|  | e) | Disc space narrowing **on a lumbar spine X-ray** correlates well with back pain | 1 | 2 | 3 |

The following five scenarios include various elements that may influence your decision to refer a patient with back pain for an X-ray. We appreciate that the scenario format means that skills you may normally draw on, such as evaluating non-verbal clues from the patient, cannot be a factor in your assessment. Nevertheless, we ask you to address *each* scenario and make a referral decision. We have left space for you to comment on any aspect of a scenario, or your decision, if you so choose.

| **2**. | The first patient is a 49 year old man. This is his third visit to the surgery. He has had a problem with his back for about 12 weeks, although he thinks it is getting better. The pain does not radiate and he has no neurological signs. He is still losing weight, but is sleeping more now. He tells you that he is not particularly worried about his condition, but he wants more of the painkillers (Co-codamol), which he thinks have been very helpful. He tells you that he really would rather not get an X-ray. | | | | | | | | | | | | | | | | | | | | |
| --- | --- | --- | --- | --- | --- | --- | --- | --- | --- | --- | --- | --- | --- | --- | --- | --- | --- | --- | --- | --- | --- |
|  | **Refer for X-ray**: | | | Yes | |  | | No | |  |  | | | |  | | | | | | |
| **On the scale 1 to 10, how difficult was it for you to make a decision for this scenario?** | | | | | | | | | | | | | | | | | | | | | |
| **Not at all** | | 0 | 1 | | 2 | | 3 | | 4 | | | 5 | 6 | 7 | | | | 8 | 9 | 10 | **Extremely difficult** |
| *If you wish to comment on this referral decision please do so here.* | | | | | | | | | | | | | | | | |  | | | | |
|  | | | | | | | | | | | | | | | |  | | | | | |
|  | | | | | | | | | | | | | | | |  | | | | | |

| **3.** | The next patient is a 30 year old woman. This is her second visit to the surgery. She has had the problem with her back for about 3 months, with the severity of the pain fluctuating over this period, sometimes getting worse with rest. She tells you she has not been right since the really bad flu she had, and that she still is occasionally feeling her temperature rise at night. She does not appear particularly worried about her condition, but she is getting very tired of the persistent pain. She tells you she would rather try some stronger painkillers than get an X-ray. | | | | | | | | | | | | | | | | | | | | |
| --- | --- | --- | --- | --- | --- | --- | --- | --- | --- | --- | --- | --- | --- | --- | --- | --- | --- | --- | --- | --- | --- |
|  | **Refer for X-ray:** | | | **Yes** | |  | | No | |  |  | | | |  | | | | | | |
| **On the scale 1 to 10, how difficult was it for you to make a decision for this scenario?** | | | | | | | | | | | | | | | | | | | | | |
| **Not at all** | | 0 | 1 | | 2 | | 3 | | 4 | | | 5 | 6 | 7 | | | | 8 | 9 | 10 | **Extremely difficult** |
| *If you wish to comment on this referral decision please do so here.* | | | | | | | | | | | | | | | | |  | | | | |
|  | | | | | | | | | | | | | | | |  | | | | | |
|  | | | | | | | | | | | | | | | |  | | | | | |

| **4.** | The 45 year old woman who comes in next is a patient of your partner who is currently on holiday. For the past 3 weeks, she has been experiencing very sharp pain in her lower back when she moves suddenly, and a dull ache the rest of the time. Your partner had prescribed painkillers 10 days before, but the problem is still persisting. You see from her notes that she had inflammatory bowel disease six months previously, which had responded well to treatment. Nevertheless, she is very worried about her symptoms and what they may mean, and feels that the problem has persisted long enough for her not to be, as she puts it, “fobbed off” with more painkillers. | | | | | | | | | | | | | | | | | | | | |
| --- | --- | --- | --- | --- | --- | --- | --- | --- | --- | --- | --- | --- | --- | --- | --- | --- | --- | --- | --- | --- | --- |
|  | **Refer for X-ray:** | | | **Yes** | |  | | No | |  |  | | | |  | | | | | | |
| **On the scale 1 to 10, how difficult was it for you to make a decision for this scenario?** | | | | | | | | | | | | | | | | | | | | | |
| **Not at all** | | 0 | 1 | | 2 | | 3 | | 4 | | | 5 | 6 | 7 | | | | 8 | 9 | 10 | **Extremely difficult** |
| *If you wish to comment on this referral decision please do so here.* | | | | | | | | | | | | | | | | |  | | | | |
|  | | | | | | | | | | | | | | | |  | | | | | |
|  | | | | | | | | | | | | | | | |  | | | | | |

| **5.** | The next patient is a 16 year old boy. He hurt his back when he was playing rugby. He had to miss the game the week after because he was still too stiff, and he hated doing that. The next week he was feeling completely better so he played, but he had to come off before the end of the game because his back was hurting him so much. It is now a week later and he is still in considerable pain. He is really anxious that this will happen every time he plays, particularly since he plans to play as a career. All movements of his lumbar spine are restricted by pain at the limits of movement. | | | | | | | | | | | | | | | | | | | | |
| --- | --- | --- | --- | --- | --- | --- | --- | --- | --- | --- | --- | --- | --- | --- | --- | --- | --- | --- | --- | --- | --- |
|  | **Refer for X-ray:** | | | **Yes** | |  | | No | |  |  | | | |  | | | | | | |
| **On the scale 1 to 10, how difficult was it for you to make a decision for this scenario?** | | | | | | | | | | | | | | | | | | | | | |
| **Not at all** | | 0 | 1 | | 2 | | 3 | | 4 | | | 5 | 6 | 7 | | | | 8 | 9 | 10 | **Extremely difficult** |
| *If you wish to comment on this referral decision please do so here.* | | | | | | | | | | | | | | | | |  | | | | |
|  | | | | | | | | | | | | | | | |  | | | | | |
|  | | | | | | | | | | | | | | | |  | | | | | |

| **6.** | The next patient is a 74 year old man. This is his first visit in relation to the shooting pain he is feeling in his back and in both legs. This episode is not the first time he has had back problems, but he is a bit hazy on the details. He says he has been experiencing the pain for a few weeks, but has put off coming to see a doctor because he hoped it would go away. He has been taking painkillers, which he thinks provide some relief. His wife made him come today. She is worried about him not getting out of the house at all because of how sore and stiff he feels. There are no neurological signs. | | | | | | | | | | | | | | | | | | | | |
| --- | --- | --- | --- | --- | --- | --- | --- | --- | --- | --- | --- | --- | --- | --- | --- | --- | --- | --- | --- | --- | --- |
|  | **Refer for X-ray:** | | | **Yes** | |  | | **No** | |  |  | | | |  | | | | | | |
| **On the scale 1 to 10, how difficult was it for you to make a decision for this scenario?** | | | | | | | | | | | | | | | | | | | | | |
| Not at all | | 0 | 1 | | 2 | | 3 | | 4 | | | 5 | 6 | 7 | | | | 8 | 9 | 10 | **Extremely Difficult** |
| *If you wish to comment on this referral decision please do so here.* | | | | | | | | | | | | | | | | |  | | | | |
|  | | | | | | | | | | | | | | | |  | | | | | |
|  | | | | | | | | | | | | | | | |  | | | | | |

| Section 5: your general style |
| --- |

| **In general, how true are the following statements**  **about you?** | | ***Not at all true*** | ***Barely***  ***true*** | ***Moderately true*** | ***Exactly***  ***true*** |
| --- | --- | --- | --- | --- | --- |
| **1.** | I can always manage to solve difficult problems if I try hard enough | 1 | 2 | 3 | 4 |
| **2.** | If someone opposes me, I can find means and ways to get what I want | 1 | 2 | 3 | 4 |
| **3.** | It is easy for me to stick to my aims and accomplish my goals | 1 | 2 | 3 | 4 |
| **4.** | I am confident that I could deal efficiently with unexpected events | 1 | 2 | 3 | 4 |
| **5.** | Thanks to my resourcefulness, I know how to handle unforeseen situations | 1 | 2 | 3 | 4 |
| **6.** | I can solve most problems if I invest the necessary effort | 1 | 2 | 3 | 4 |
| **7.** | I can remain calm when facing difficulties because I can rely on my coping abilities | 1 | 2 | 3 | 4 |
| **8.** | When I am confronted with a problem, I can usually find several solutions | 1 | 2 | 3 | 4 |
| **9.** | If I am in a bind, I can usually think of something to do | 1 | 2 | 3 | 4 |
| **10.** | No matter what comes my way, I’m usually able to handle it | 1 | 2 | 3 | 4 |

# Consent Slip

Please answer the following questions:

|  | |  |  | Please mark the appropriate box | | | |
| --- | --- | --- | --- | --- | --- | --- | --- |
| I give permission for the research team to obtain the number of referrals that I have made to the Radiology Departments listed below between August 2001 | | |  | | |  |  |
|  | and January 2005 | |  | | | | |
|  | | | | |  | | |
| I do not wish to participate in the project | | |  | | | | |
| Please tick the following box if you agree to participate in the project and would like to receive a summary of the results arising from this project | |  |  | | |  |  |
|  | |  |  | | |  |  |

**Which hospitals do you refer patients for a lumbar spine X-ray? Please estimate approximately how many of these referrals would go to each hospital if you refer to more than one hospital.**

|  | **Hospital** | **Approximate proportion**  **of referrals** |
| --- | --- | --- |
|  |  |  |
|  |  |  |
|  |  |  |
|  |  |  |
|  |  |  |
|  |  |  |

**Are any of the lumbar spine X-rays that your hospital reports in your name actually ordered by someone else, such as a vocational training scheme registrar, an assistant or a locum?**

| **Yes** |  | **No** |  |
| --- | --- | --- | --- |

If yes, could you estimate the proportion over the last twelve months %

Signature…………………………. Date….../….../……...

*Please return the completed questionnaire and consent form in the enclosed pre-paid envelope.*

*We would like to thank you for your participation in this study - your contribution is very much appreciated.*
