# Supplementary material for: Applying psychological theories to evidence-based clinical practice: identifying factors predictive of lumbar spine x-ray for low back pain in UK primary care practice
Source: Implement Sci. 2011 May 28;6:55. doi: 10.1186/1748-5908-6-55 (PMC3125229; doi:10.1186/1748-5908-6-55)
Supplement: Additional File 2 — Goodness of fit models for the negative binomial regression analysis [file 1748-5908-6-55-S2.PDF]

Appendix: Goodness of fit models for the negative binomial regression analysis

In linear regression the R^2 statistic has an intuitive interpretation of the proportion total variation in outcome that is accounted for by that model. So for example in the main paper in **Table 3** the adjusted R^2 statistic for The Theory of Planned Behaviour (TPB) predicting intention was 0.25. This means that 25% of the variation in intention was accounted for by this model. Also in that table Social Cognitive Theory (SCT) accounted for the 12.1% of the variation in behavioural simulation. The adjusted R^2 statistic allows us to compare these different models on different outcomes directly and we can make the statement that TPB was better than explaining the variation in intention than SCT is for behavioural simulation.

When modelling using negative binomial regression a directly analogous R^2 is not available and such comparisons are not possible. However, there do exist measures of fit that can be used to assess competing models on the same outcome in the same dataset. There are a range of psudeo-R^2 and other fit statistics that qualitatively do the same thing a R^2 in linear regression. The Stata post-estimation command `fitstat` produces measure of fit statistics for negative binomial regression models and was used to generate the statistics tabulated below. The pseudo-R^2 we chose to use was McFaddens' adjusted R^2 because it penalises models in the spirit of adjusted R^2 in linear regression for adding more variables to a model. Negative values are possible for McFaddens' adjusted R^2 but we have truncated these to be zero. Note that a pseudo-R^2 can only be compared to another pseudo-R^2 of the same type.

On the page below there is a table of summary statistics for the TPB negative binomial regression model used to predict behaviour. The command `fitstat` compared the current model (TPB) to a saved model. The saved model was the null model, that is an empty model that contains only

the intercept and is the basic model that we compared all the theoretical models to. For TPB the McFaddens' adjusted R^2 was 0.004 which is close to zero. The other reported pseudo-R^2 statistics support this finding. Information Criteria statistics such as Akaike's Information Criteria (AIC) and the Bayesian Information Criteria (BIC) suggest that given the complexity of the theoretical model TPB does not explain any useful variation over the null model. However the likelihood statistics suggest the TPB may be useful model for this dataset, and this is reflected in the coefficient for PBC power being significant. This discrepancy can be partially explained by the large sample size used to estimate the model (upon which significance tests depend). Summarising all the information it would suggest evidence that TPB had limited predictive ability for the behaviour as measured in this dataset.

For a detailed description of the statistics below see *Freese, Jeremy and J. Scott Long. Regression Models for Categorical Dependent Variables Using Stata. College Station: Stata Press, 2006.*

Theory of Planned Behaviour

	Current	Saved	Difference
N:	240	240	0
Log-Lik Intercept Only	-669.489	-669.489	0.000
Log-Lik Full Model	-661.507	-669.489	7.982
D	1323.014(235)	1338.978(238)	15.965(3)
LR	15.965(3)	0.000(0)	15.965(3)
Prob > LR	0.001	.	0.001
McFadden's R2	0.012	0.000	0.012
McFadden's Adj R2	0.004	-0.003	0.007
ML (Cox-Snell) R2	0.064	0.000	0.064
Cragg-Uhler(Nagelkerke) R2	0.065	0.000	0.065
AIC	5.554	5.596	-0.042
AIC*n	1333.014	1342.978	-9.965
BIC	35.064	34.586	0.477
BIC'	0.477	-0.000	0.477
BIC used by Stata	1350.417	1349.940	0.477
AIC used by Stata	1333.014	1342.978	-9.965

Social Cognitive Theory

	Current	Saved	Difference
N:	240	240	0
Log-Lik Intercept Only	-669.489	-669.489	0.000
Log-Lik Full Model	-662.251	-669.489	7.238
D	1324.502(234)	1338.978(238)	14.476(4)
LR	14.476(4)	0.000(0)	14.476(4)
Prob > LR	0.006	.	0.006
McFadden's R2	0.011	0.000	0.011
McFadden's Adj R2	0.002	-0.003	0.005
ML (Cox-Snell) R2	0.059	0.000	0.059
Cragg-Uhler(Nagelkerke) R2	0.059	0.000	0.059
AIC	5.569	5.596	-0.027
AIC*n	1336.502	1342.978	-6.476
BIC	42.033	34.586	7.446
BIC'	7.446	-0.000	7.446
BIC used by Stata	1357.386	1349.940	7.446
AIC used by Stata	1336.502	1342.978	-6.476

Implementation Intention

	Current	Saved	Difference
N:	240	240	0
Log-Lik Intercept Only	-669.489	-669.489	0.000
Log-Lik Full Model	-668.318	-669.489	1.172
D	1336.635(237)	1338.978(238)	2.343(1)
LR	2.343(1)	0.000(0)	2.343(1)
Prob > LR	0.126	.	0.126
McFadden's R2	0.002	0.000	0.002
McFadden's Adj R2	-0.003	-0.003	0.000
ML (Cox-Snell) R2	0.010	0.000	0.010
Cragg-Uhler(Nagelkerke) R2	0.010	0.000	0.010
AIC	5.594	5.596	-0.001
AIC*n	1342.635	1342.978	-0.343
BIC	37.724	34.586	3.138
BIC'	3.138	-0.000	3.138
BIC used by Stata	1353.077	1349.940	3.138
AIC used by Stata	1342.635	1342.978	-0.343

Operant Learning Theory

	Current	Saved	Difference
N:	240	240	0
Log-Lik Intercept Only	-669.489	-669.489	0.000
Log-Lik Full Model	-662.972	-669.489	6.517
D	1325.944(236)	1338.978(238)	13.035(2)
LR	13.035(2)	0.000(0)	13.035(2)
Prob > LR	0.001	.	0.001
McFadden's R2	0.010	0.000	0.010
McFadden's Adj R2	0.004	-0.003	0.007
ML (Cox-Snell) R2	0.053	0.000	0.053
Cragg-Uhler(Nagelkerke) R2	0.053	0.000	0.053
AIC	5.558	5.596	-0.038
AIC*n	1333.944	1342.978	-9.035
BIC	32.513	34.586	-2.073
BIC'	-2.073	-0.000	-2.073
BIC used by Stata	1347.866	1349.940	-2.073
AIC used by Stata	1333.944	1342.978	-9.035

Common Sense Self-regulation Model

	Current	Saved	Difference
N:	240	240	0
Log-Lik Intercept Only	-669.489	-669.489	0.000
Log-Lik Full Model	-654.551	-669.489	14.938
D	1309.102(222)	1338.978(238)	29.877(16)
LR	29.877(16)	0.000(0)	29.877(16)
Prob > LR	0.019	.	0.019
McFadden's R2	0.022	0.000	0.022
McFadden's Adj R2	-0.005	-0.003	-0.002
ML (Cox-Snell) R2	0.117	0.000	0.117
Cragg-Uhler(Nagelkerke) R2	0.117	0.000	0.117
AIC	5.605	5.596	0.009
AIC*n	1345.102	1342.978	2.123
BIC	92.400	34.586	57.813
BIC'	57.813	-0.000	57.813
BIC used by Stata	1407.753	1349.940	57.813
AIC used by Stata	1345.102	1342.978	2.123

Precaution Adoption Process

	Current	Saved	Difference
N:	240	240	0
Log-Lik Intercept Only	-675.532	-669.489	-6.042
Log-Lik Full Model	-675.292	-669.489	-5.803
D	1350.585(237)	1338.978(238)	11.606(1)
LR	0.479(1)	0.000(0)	0.479(1)
Prob > LR	0.489	.	0.489
McFadden's R2	0.000	0.000	0.000
McFadden's Adj R2	-0.004	-0.003	-0.001
ML (Cox-Snell) R2	0.002	0.000	0.002
Cragg-Uhler(Nagelkerke) R2	0.002	0.000	0.002
AIC	5.652	5.596	0.057
AIC*n	1356.585	1342.978	13.606
BIC	51.673	34.586	17.087
BIC'	5.002	-0.000	5.002
BIC used by Stata	1367.026	1349.940	17.087
AIC used by Stata	1356.585	1342.978	13.606

Knowledge

	Current	Saved	Difference
N:	240	240	0
Log-Lik Intercept Only	-675.532	-669.489	-6.042
Log-Lik Full Model	-674.913	-669.489	-5.424
D	1349.826(237)	1338.978(238)	10.847(1)
LR	1.238(1)	0.000(0)	1.238(1)
Prob > LR	0.266	.	0.266
McFadden's R2	0.001	0.000	0.001
McFadden's Adj R2	-0.004	-0.003	-0.001
ML (Cox-Snell) R2	0.005	0.000	0.005
Cragg-Uhler(Nagelkerke) R2	0.005	0.000	0.005
AIC	5.649	5.596	0.054
AIC*n	1355.826	1342.978	12.847
BIC	50.914	34.586	16.328
BIC'	4.243	-0.000	4.243
BIC used by Stata	1366.268	1349.940	16.328
AIC used by Stata	1355.826	1342.978	12.847
